# Recurrent high creatine kinase levels under clozapine treatment - a case report assessing a suspected adverse drug reaction

**DOI:** 10.3389/fpsyt.2024.1397876

**Published:** 2024-04-29

**Authors:** Florine M. Wiss, Samuel S. Allemann, Henriette E. Meyer zu Schwabedissen, Céline K. Stäuble, Thorsten Mikoteit, Markus L. Lampert

**Affiliations:** ^1^ Pharmaceutical Care, Department of Pharmaceutical Sciences, University of Basel, Basel, Switzerland; ^2^ Institute of Hospital Pharmacy, Solothurner Spitäler, Olten, Switzerland; ^3^ Biopharmacy, Department of Pharmaceutical Sciences, University of Basel, Basel, Switzerland; ^4^ Psychiatric Services Solothurn, Solothurner Spitäler and Department of Medicine, University of Basel, Solothurn, Switzerland

**Keywords:** therapeutic drug monitoring, pharmacogenetic, adverse drug reaction, adherence, clozapine, CYP2D6, CYP3A5

## Abstract

Suspected adverse drug reactions (ADRs) during treatment with clozapine often prompt therapeutic drug monitoring (TDM) in clinical practice. Currently, there is no official recommendation for pharmacogenetic (PGx) testing in the context of clozapine therapy. In this case report, we demonstrate and discuss the challenges of interpreting PGx and TDM results highlighting the possibilities and limitations of both analytical methods. A 36-year-old male patient with catatonic schizophrenia was treated with clozapine. He experienced multiple hospitalizations due to elevated creatine kinase (CK) levels (up to 9000 U/L, reference range: 30-200 U/L). With no other medical explanation found, physicians suspected clozapine-induced ADRs. However, plasma levels of clozapine were consistently low or subtherapeutic upon admission, prompting us to conduct a PGx analysis and retrospectively review the patient’s TDM data, progress notes, and discharge reports. We investigated two possible hypotheses to explain the symptoms despite low clozapine plasma levels: *Hypothesis i.* suggested the formation and accumulation of a reactive intermediate metabolite due to increased activity in cytochrome P450 3A5 and reduced activity in glutathione S-transferases 1, leading to myotoxicity. *Hypothesis ii.* proposed under-treatment with clozapine, resulting in ineffective clozapine levels, leading to a rebound effect with increased catatonic symptoms and CK levels. After considering both data sources (PGx and TDM), hypothesis ii. appeared more plausible. By comprehensively assessing all available TDM measurements and examining them in temporal correlation with the drug dose and clinical symptoms, we observed that CK levels normalized when clozapine plasma levels were raised to the therapeutic range. This was achieved through hospitalization and closely monitored clozapine intake. Therefore, we concluded that the symptoms were not an ADR due to altered clozapine metabolism but rather the result of under-treatment. Interpreting TDM and PGx results requires caution. Relying solely on isolated PGx or single TDM values can result in misinterpretation of drug reactions. We recommend considering the comprehensive patient history, including treatment, dosages, laboratory values, clinic visits, and medication adherence.

## 1 Introduction

Clozapine is considered one of the most effective neuroleptics for patients with treatment-resistant schizophrenia, but it is associated with severe and potentially life-threatening adverse drug reactions (ADRs). Therefore, clozapine should only be used for patients who did not respond to at least two different antipsychotics, including a second-generation antipsychotic medication (1, 2). One of the rare but most feared side effects of clozapine is agranulocytosis (3). While clozapine-induced agranulocytosis is assumed to be independent of dose, many dose-dependent adverse effects, like seizures, metabolic syndrome, and constipation, are known (4, 5). Another rare but less known ADR of clozapine is myotoxicity, which may be associated with elevated creatine kinase (CK) levels (6, 7). However, the mechanism behind the development of this ADR is poorly understood (8).

The efficacy of clozapine as well as its dose-dependent adverse effects correlate with clozapine plasma levels (5). Efficacy depends on reaching therapeutic plasma concentration with a recommended level of at least 0.35 mg/L in treatment-resistant schizophrenia (9). Dose-dependent ADRs are assumed to be associated with plasma levels above 0.60 mg/L (5). The narrow therapeutic window suggests that clozapine efficacy and safety could be very sensitive to changes in plasma concentration (5). Accordingly, guidelines recommend therapeutic drug monitoring (TDM) for dose titration and in cases of suspected ADRs, clinical non-response, suspected drug-drug interactions, or suspected non-adherence (1, 2, 9).

However, clozapine plasma levels show high interindividual variability (5). Patient-related factors that influence clozapine plasma concentrations include gender, age, body weight, inflammation, and smoking behavior (10, 11). Smoking, in particular, can affect plasma levels by inducing cytochrome P450 1A2 (CYP1A2) and thereby increasing clozapine metabolism (12, 13).

Another factor that could affect clozapine plasma levels, efficacy, and safety is pharmacogenetics (PGx) (14). PGx investigates genetic variations in drug metabolizing enzymes, drug transporters, and drug targets and relates them to drug response (15). For example, genetic variants play a substantial role in the aforementioned inducibility of CYP1A2 by smoking. Individuals with a CYP1A2*1F genotype, for instance, exhibit increased inducibility by smoking, which has been linked to treatment failure of clozapine in schizophrenia patients (16, 17).

Unlike TDM, PGx is rarely performed in routine practice when assessing clozapine therapy failure. Nevertheless, because of its narrow therapeutic window and its hepatic metabolism, clozapine is assumed to be a drug whose efficacy and safety profile would benefit from a PGx-oriented TDM, including both traditional TDM findings and PGx analyses (18).

In this case report, we present a patient with elevated CK levels during clozapine therapy. We collected and interpreted both PGx and TDM data to understand the underlying cause of the potential clozapine-induced ADR. We aim to demonstrate and discuss the challenges of interpreting PGx and TDM results highlighting the possibilities and limitations of both analytical methods.

## 2 Case presentation

We report the case of a 36-year old male patient of Central American descent diagnosed with catatonic schizophrenia. Catatonic schizophrenia is a subtype of schizophrenia characterized by prominent psychomotor disorders, such as periods of immobility or rigidity, as well as episodes of severe agitation (19). The patient has been treated with clozapine since 2016 due to insufficient response to multiple other neuroleptics. His drug history includes aripiprazole, olanzapine, promazine, and risperidone.

In the end of May 2022, the patient was hospitalized for confusion and subjective weakness. Laboratory tests revealed markedly elevated CK levels (3,787 U/L; reference range: 30-200 U/L), prompting admission to the intensive care unit (ICU). After 4 days CK levels continued to rise (peaking at 8,999 U/L) and ICU physicians feared a clozapine-induced malignant neuroleptic syndrome (MNS) and promptly discontinued clozapine therapy. However, apart from the elevated CK levels, no other symptoms suggestive of MNS were observed. The patient was afebrile, normocytic, showed normal heart rate, no signs of autonomic dysregulation, and had no rigidity. Differential diagnosis ruled out other causes of rhabdomyolysis, such as infections, traumas, and electrolyte imbalances. Additionally, the toxicology screening was negative. Due to severe restlessness, verbal hallucinations, and delusional fears, the patient was treated with lorazepam (fixed and as needed) and dexmedetomidine via infusion. Additionally, the patient was hydrated. During the ICU stay, therapeutic drug monitoring (TDM) of clozapine was initiated. The measured clozapine plasma level of 0.156 mg/L at a daily dose of 200 mg was below the therapeutic reference range (therapeutic reference range: 0.35-0.60 mg/L) and within the expected dose-dependent clozapine reference range (0.09-0.32 mg/L). Further laboratory values measured during the ICU stay are shown in the **Supplementary Table 1**. In addition to elevated CK levels, aspartate aminotransferase (ASAT) values exceeded the upper limit of normal (ULN) by more than three times.

Of note, this was not the patient’s first hospitalization due to elevated CK levels. The patient had been hospitalized multiple times for the same reason in previous years. On each occasion, clozapine was suspected as the potential cause for the elevated CK levels. However, the measured plasma levels of clozapine were often low or even subtherapeutic upon clinic admission. Furthermore, clozapine was continued and well tolerated between ICU admissions, reducing the likelihood that the CK elevation was due to MNS. This raised the question of whether clozapine could truly be attributed as the trigger for the CK elevation. To investigate this matter, the physicians consulted the clinical pharmacy department at the hospital.

Searching for a possible explanation for suspected drug-induced ADRs despite low plasma levels, the clinical pharmacists postulated two hypotheses (hypothesis *i.* and hypothesis *ii.*).

### 2.1 Hypotheses

#### 2.1.1 Hypothesis i. Altered clozapine metabolism and the generation of potentially toxic metabolites

**Figure 1:**
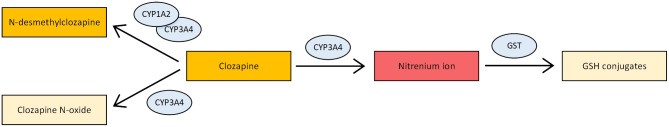
Hepatic metabolism of clozapine: pharmacological active compounds are colored dark orange, inactive compounds light orange, and toxic compounds red.

Clozapine undergoes extensive hepatic metabolism (**Figure 1**), with the major pathways being demethylation to N-desmethylclozapine (an active metabolite) and oxidation to clozapine N-oxide (20). CYP1A2 and CYP3A4 are primarily responsible for catalyzing the demethylation pathway, while CYP3A4 is responsible for the N-oxidation pathway (21, 22). In addition to the formation of these stable metabolites, CYP3A4 is thought to be involved in the formation of a reactive intermediate metabolite (Nitrenium ion), which may lead to certain clozapine ADRs, such as hepatotoxicity (23) or myotoxicity (7). Several human Glutathione S-Transferases (GSTs) are responsible for the detoxification of this metabolite, with GSTP1 exhibiting the highest activity (24). Other enzymes, such as CYP2C19, CYP2D6, or flavin-containing monooxygenase 3 (FMO3), have been linked to clozapine metabolism, whereas their role in clozapine metabolism seems to be less clear (23, 25). However, CYP2C19 appears to play also a relevant role in metabolism, as it has been demonstrated that CYP2C19 poor metabolizers (PM) exhibit increased clozapine plasma levels compared to extensive metabolizers (EM) (26).

Based on this understanding of clozapine metabolism, we hypothesized that genetic variants altering clozapine metabolism could potentially contribute to clozapine toxicity. Consequently, we initiated a PGx analysis for this patient in September 2022.

For PGx analysis, the patient was included in an observational study at our hospital (ClinicalTrials.gov identifier: NCT04154553) approved by the local ethics committee (EKNZ ID: 2019-01452). After obtaining informed consent, we performed panel pharmacogenotyping, applying the commercial service Stratipharm^®^ offered by humatrix AG (Pfungstadt, Germany). This analysis includes 30 genes encoding for transport proteins, metabolizing enzymes, and drug targets.

#### 2.1.2 Hypothesis ii. Clozapine under-treatment resulting in increased catatonic symptoms including CK elevation

Because of the severe psychomotor symptoms, patients with catatonic schizophrenia often have slightly elevated CK levels compared to healthy individuals or psychiatric patients without catatonia. Northoff et al. (27) reported a mean CK level of around 320 U/L in 17 patients with catatonic schizophrenia.

Based on this observation, we hypothesized that under-treatment with clozapine might lead to increased catatonic symptoms and that increased catatonic symptoms might consequently lead to elevated CK levels. Consequently, we conducted a thorough review of the patient’s data from August 2020 to September 2022. The patient’s medical history was analyzed based on TDM data, medical notes, and reports gathered from hospitalizations in somatic clinics, as well as from various psychiatric in-patient treatments.

### 2.2 Results and interpretation

#### 2.2.1 PGx assessment (i)

The identified variants in the PGx panel test identified the patient as a CYP2C19 poor metabolizer (PM, *2 homozygous) and CYP2D6 intermediate metabolizer (IM, *29 homozygous). CYP3A5 showed increased activity (IM, *3 heterozygous), whereas the predicted phenotype for CYP1A2 showed increased inducibility (*1F heterozygous) (**Table 1**). Additionally, for GSTP1 the patient was identified heterozygote for rs1695 (*GSTP1-313AG*).

**Table 1:** Selected results of panel-pharmacogenotyping and phenotype interpretation.

Gene	Variant *(also tested variants in gen locus)*	Genotype	Diplotype	Predicted phenotype
CYP1A2	rs762551 g.75041917C>A (in *1F) *(rs2069514)*	C/A	*1A/*1F	increased inducibility
CYP2C19	rs4244285 c.681G>A (in *2) *(rs4986893, rs12248560, rs28399504)*	A/A	*2/*2	poor metabolizer *(no function)*
CYP2D6	rs59421388 c.1012G>A (in *29) *(CNV^a^, rs35742686, rs3892097, rs5030655, rs5030867, rs5030865, rs5030656, rs1065852, rs201377835, rs28371706, rs28371725)*	A/A	*29/*29	intermediate metabolizer *(AS^b^ = 1, reduced function)*
CYP3A5	rs776746 c.219-237G>A (in*3)	A/G	*1/*3	intermediate metabolizer *(increased function compared to major phenotype)*
GSTP1	rs1695 c.313A>G	A/G	n.d.^c^	n.d.^c^ *(substance specific)*

^a^CNV, copy number variation; ^b^AS, activity score; ^c^n.d., not defined.

Based on the genetic profile, we suspected altered clozapine metabolism due to no enzyme activity in CYP2C19 (PM phenotype), induced activity in CYP1A2 (combined effect due to CYP1A2 *1F allele and induction by smoking) and increased activity in CYP3A5 (CYP3A5*1/*3 genotype). Interestingly, most Europeans are homozygous variants in CYP3A5 (*3/*3), which results in no enzyme expression in adults. Less than 10% of all Caucasians show a *1 allele with the expression of CYP3A5. Conversely, among people of African-American descent, the *1 allele is significantly more prevalent, with approximately 60-70% of individuals carrying this allele (28, 29). Since CYP3A5 has a similar substrate spectrum as CYP3A4 (30) and CYP3A5*1 genotype has been shown to alter clozapine metabolism in some studies (31, 32), an increased formation of the reactive intermediate toxic metabolite by CYP3A5 is conceivable. Additionally, the patient showed a heterozygous genotype for the rs1695 in *GSTP1*, which is associated with reduced enzyme activity (33). We concluded that increased activity in CYP3A5 and reduced activity in GSTP1 might have contributed to the accumulation of the reactive intermediate metabolite, providing a potential explanation for the toxicity despite of low plasma levels observed in this case.

#### 2.2.2 Patient history and TDM assessment (ii)

**Figure 2:**
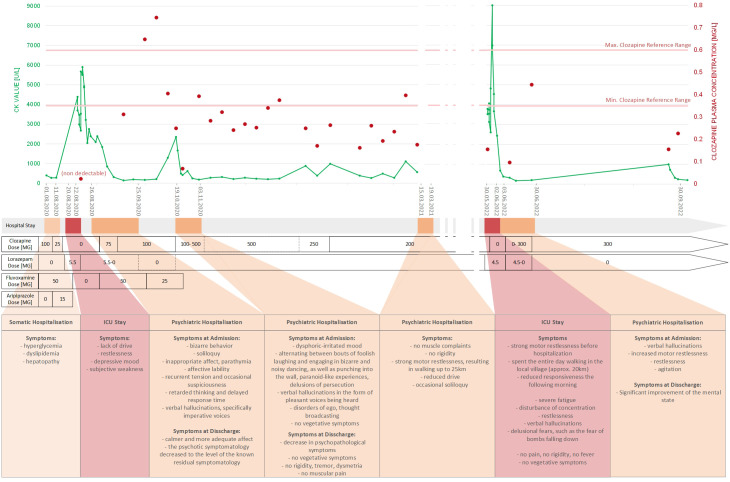
Summary of medical history from August 2020 to September 2022.

The collected TDM data and information from medical notes, and reports are summarized in **Figure 2**, providing an overview of the patient’s complete medical history from August 2020 to September 2022. Additionally, we present a selection of measured clozapine and N-desmethyl clozapine plasma levels along with their corresponding expected plasma levels at a specific clozapine dose in **Table 2**. We specifically chose these measurements as they were obtained during a psychiatric in-patient treatment, allowing us to monitor uninterrupted medication intake and steady-state measurements. The expected plasma levels were calculated following the instructions in the *Consensus Guidelines for Therapeutic Drug Monitoring in Neuropsychopharmacology* (34).

**Table 2:** A selection of measured clozapine and N-desmethyl clozapine plasma levels at steady-state and controlled medication intake in comparison with the expected, dose-dependent reference range of clozapine and N-desmethyl clozapine.

Date	Clozapine Daily Dose [mg]	Measured Clozapine Plasma Level [mg/L] (Therapeutic reference 0.35-0.6 mg/L)^34^	Expected dose-dependent Clozapine reference range [mg/L]^a^	Measured N-desmethyl clozapine Plasma Level [mg/L]	Expected dose-dependent N-desmethyl clozapine reference range [mg/L]^a^	N-desmethyl clozapine/Clozapine *(Reference 0.45-0.79)* ^34^
17.09.2020	75	0.311^b^	0.03-0.12	0.050^b^	0.037-0.093	0.161^b^
02.11.2020	500	0.393	0.22-0.80	0.230	0.250-0.625	0.585
27.06.2022	300	0.445	0.13-0.48	0.143	0.150-0.380	0.321

a Calculated according to the Consensus Guidelines for Therapeutic Drug Monitoring in Neuropsychopharmacology^34^.

b Clozapine in combination with the CYP inhibitor fluvoxamine (50mg).

Overall, the data revealed a recurrent pattern: since August 2020 the patient has been hospitalized several times due to increased CK levels. Prior to hospitalization, the patient experienced severe psychotic and catatonic symptoms, including agitation, verbal hallucinations, self-talk, aggression, and a strong motor restlessness. Before being hospitalized, the patient occasionally walked long distances per day (20-25 kilometers). Upon admission to the hospital, physicians discovered elevated CK levels, which led to ICU treatment in two cases (August 2020 and May 2022). Suspecting ADRs such as MNS, clozapine was discontinued, although TDM analysis showed subtherapeutic levels upon clinic entry in each case. After being treated in the ICU (lorazepam, dexmedetomidine and hydration), the patient was referred to a psychiatric follow-up in a psychiatric clinic. During inpatient psychiatric treatment, clozapine therapy was restarted and increased in dose due to severe psychotic symptoms. Clozapine is chosen because there is evidence in the literature that clozapine is considered superior to other neuroleptics in the treatment of catatonic schizophrenia with respect to psychiatric and catatonic symptoms (35). Under controlled medication intake, the clozapine plasma levels reached the therapeutic reference range, and the psychotic and catatonic symptoms, as well as CK levels, normalized in each case. Interestingly, the patient denied muscle discomfort or pain at all times.

Based on the chronological documentation of TDM data, we observed a correlation between subtherapeutic plasma levels and increased CK levels. Several causes were identified for subtherapeutic plasma levels: In August 2020, clozapine was discontinued due to a newly diagnosed clozapine-induced hepatopathy. In addition to hepatopathy, the patient also showed hyperglycemia and dyslipidemia. Clozapine was discontinued and replaced by aripiprazole. Five days later, the patient was admitted with exacerbated psychiatric symptoms and elevated CK levels leading to ICU stay.

Another reason for subtherapeutic plasma levels was a low daily dosage of clozapine that had been prescribed for a considerable period of time. Due to the recurrent elevation of CK levels and the assumption of dose-related ADRs, psychiatrists reduced clozapine dosage from 500mg to 200mg daily in January 2021. Throughout the entirety of 2021, the patient received 200 mg of clozapine daily, under which his psychiatric and catatonic symptoms were poorly controlled. Nevertheless, the psychiatrists no longer dared to increase the dosage of clozapine to the patient’s effective dose. Furthermore, we suspected poor medication adherence to be an additional issue. The patient admitted to occasionally forgetting to take his pills.

Due to this observed correlation between low plasma levels and increased CK levels, we concluded that, in this case, elevated CK levels were not the consequence of clozapine overdosing. On the contrary, clozapine under-treatment seemed to be responsible for the increase in CK levels. Interestingly, there is evidence in the literature that abrupt clozapine withdrawal may lead to a rebound phenomenon with increased catatonic symptoms. There is a hypothesis that extended use of clozapine enhances gamma-aminobutyric acid (GABA) activity, and discontinuing it may heighten excitatory neurotransmission, potentially resulting in catatonia (19). Several cases of withdrawal catatonia after clozapine discontinuation have been described in the literature (36). In one case the catatonic symptoms were associated with a substantial CK elevation (up to 2400 U/L) (37).

### 2.3 Conclusion and outcome

Based on the PGx and TDM data, two hypotheses have been proposed to explain the clozapine-induced CK elevation:

*(i)* Increased activity in CYP3A5 and reduced activity in GSTP1 contribute to the accumulation of a reactive intermediate metabolite responsible for myotoxicity.
*(ii)* Under-treatment with clozapine results in ineffective clozapine levels leading to a rebound effect with increased catatonic symptoms leading to elevated CK levels.

After considering all available information (PGx and TDM), the second hypothesis *(ii)* appeared to be more plausible than the first hypothesis *(i).* Hypothesis*(ii)* was supported by the time correlation between low clozapine plasma levels and CK increase, as well as the fact that controlled drug use during psychiatric hospitalization and clozapine plasma levels within the therapeutic reference range led to a reduction in CK levels. Additionally, pharmacokinetic calculations and considerations based on the TDM data did not support hypothesis *(i).* The measured clozapine levels including the N-desmethylclozapine/clozapine ratio were mostly within the expected reference range for the respective daily dose. These observations do not support altered clozapine metabolism caused by any genetic variants, making the hypothesis of toxic metabolite accumulation unlikely. An altered clozapine metabolism was only detected when clozapine was combined with fluvoxamine, a potent inhibitor of CYP1A2 known to influence clozapine metabolism (August-October 2020, see **Figure 2**; **Table 2**) (38).

Based on these findings, we concluded that maintaining therapeutic levels of clozapine is necessary to prevent withdrawal catatonia and CK elevations in this case. This requires prescribing a sufficiently high dose of clozapine and ensuring strict adherence by the patient. As a result, the psychiatrists prescribed a daily clozapine dose of 300 mg and implemented a controlled drug regimen with supervised medication intake in September 2022. Since then, up to March 2024, the patient has remained stable and has not required further hospitalization due to increased CK levels.

## 3 Discussion

Clozapine efficacy and plasma levels can vary significantly between individuals. Due to this variability, TDM is recommended for clozapine therapy and is frequently performed in clinical practice (5). In contrast, there is no official recommendation for PGx testing with regard to clozapine therapy (39). It is controversial whether TDM analyses are sufficient or whether PGx testing could complement them (18).

In our case, the TDM analysis proved valuable in uncovering a correlation between low plasma levels and an increase in CK levels. This finding was only possible by comprehensively assessing all available TDM measurements and examining them in temporal correlation with the drug dose, the corresponding expected serum drug levels, and clinical symptoms. It is essential to avoid misinterpretation by evaluating single TDM values, especially when they are not steady-state readings (40). Furthermore, TDM data are meaningful only if continuous drug intake is ensured, therefore steady-state plasma concentrations are reached, and interactions with potential CYP inducers or inhibitors (such as fluvoxamine) are taken into account (5, 41).

The observation that clozapine discontinuation and under-treatment led to increased CK levels highlights the fact that drug related problems are not always a result of high drug levels and drug toxicity. Especially withdrawal symptoms can occur after abrupt discontinuation of psychiatric medications such as clozapine (36). However, the dose-dependent understanding of drug-related problems often leads to a tendency to discontinue drugs or reduce dosages when unexpected drug reactions are suspected. In our case as well, clozapine was discontinued each time the patient was admitted to the ICU. However, it should be noted that making a correct diagnosis of elevated CK levels was challenging in this case. ICU physicians had only limited knowledge about the patient’s history and based their decisions solely on the current clinical presentation. This made differentiation between various forms of catatonia, such as malignant catatonia, MNS, and withdrawal catatonia, difficult. Although these catatonia forms have different origins, they present with very similar symptoms (19). Given the high mortality associated with MNS, discontinuing Clozapine upon suspicion of MNS was the correct decision in this situation. The conclusion that the elevated CK levels were due to withdrawal catatonia was only possible retrospectively, once all patient data were gathered, and the temporal correlation between Clozapine undertreatment and CK elevation was observed. Therefore regarding clozapine, more informed decisions should be made when suspecting clozapine-related problems, taking into account that abrupt discontinuation of clozapine can result in withdrawal catatonia (36).

With this case, we demonstrate that interpreting PGx results regarding clozapine must be done with caution. Relying solely on considerations of drug metabolism and pharmacokinetic models can lead to misinterpretations. It is essential to base the interpretation of PGx results on evidence-based recommendations provided by PGx working groups such as the Clinical Pharmacogenetics Implementation Consortium (CPIC) or the Dutch Pharmacogenetics Working Group (DPWG). These recommendations are based on a systematic literature search and evidence assessment. In fact, the latest DPWG guidelines do not recommend adjusting clozapine doses based on genetic variants in *CYP2D6, CYP1A2* or *CYP3A5* (39). However, the DPWG guidelines do provide recommendations for dose reduction in individuals with PM status in CYP2D6 when using aripiprazole and risperidone (39). This suggests that PGx analyses could provide valuable insights for patients with schizophrenia, aiding in the optimization of their pharmacotherapy. But it’s important to note, as demonstrated in this case report, that PGx data should never be considered in isolation. Instead, it should always be evaluated in the broader context, considering factors like concomitant medication, symptoms, patient history, medication adherence and additional laboratory data, such as TDM.

Although PGx could not provide an explanation for the suspected ADR to clozapine in our case, further research in this area would be of interest. There are reports in the literature of CK elevation during clozapine therapy in patients with schizophrenia without catatonic symptoms (8). It is possible that genetic factors may play a role in these cases, and further investigation may yield insights into the underlying mechanisms.

## 4 Conclusion

This case report underscores the significance of a systematic evaluation of the patient’s history when assessing suspected ADRs. It involves integrating all available information, including symptoms, indication history, TDM measurements, and PGx data, and correlating them temporally. In this instance, the suspected clozapine-induced ADR was more likely attributed to under-treatment with clozapine. This conclusion was made possible through a comprehensive evaluation of all available TDM measurements, which were carefully examined in temporal correlation with the drug dose and clinical symptoms.

Regarding PGx information to guide clozapine therapy, caution is warranted as the existing literature on this topic remains contradictory, making it challenging to provide recommendations. However, it is conceivable that in the future, both TDM and PGx results could be considered in guiding clozapine therapy and interpreting ADRs, enhancing our understanding of individual responses to the medication.

## Data availability statement

The original contributions presented in the study are included in the article/**Supplementary Material**. Further inquiries can be directed to the corresponding author.

## Ethics statement

The studies involving humans were approved by Ethikkommission Nordwest- und Zentralschweiz (EKNZ ID: 2019-01452). The studies were conducted in accordance with the local legislation and institutional requirements. The participants provided their written informed consent to participate in this study. Written informed consent was obtained from the individual(s) for the publication of any potentially identifiable images or data included in this article.

## Author contributions

FW: Conceptualization, Data curation, Investigation, Visualization, Writing – original draft. SA: Conceptualization, Writing – review & editing. HM: Investigation, Writing – review & editing. CS: Conceptualization, Investigation, Writing – review & editing. TM: Investigation, Writing – review & editing. ML: Conceptualization, Supervision, Writing – review & editing, Investigation.
