# Supplementary material for: Recurrent high creatine kinase levels under clozapine treatment - a case report assessing a suspected adverse drug reaction
Source: Front Psychiatry. 2024 Apr 29;15:1397876. doi: 10.3389/fpsyt.2024.1397876 (PMC11089194; doi:10.3389/fpsyt.2024.1397876)
Supplement: Supplementary file 1 [file Table_1.docx]

Supplementary table 1. Selected laboratory values measured during ICU stay in 2022

| **Lab Value [Reference Range]** | **Measurement from 02.06.2022** |
| --- | --- |
| Clozapine Plasma Level [0.35-0.60 mg/L] | 0.156 mg/L |
| CK [30-200 U/L] | 8,999 U/L |
| Leukocytes [3.9-9.5 *10^9^L] | 8.9 *10^9^L |
| ASAT [5-35 U/L] | 135 U/L |
| ALAT [< 45 U/L] | 55 U/L |
| Troponin I hs [< 26.3 ng/L] | < 4.0 ng/L |
| CRP [< 5.1 mg/L] | 5.4 mg/L |

CK creatine kinase; ASAT Aspartate Aminotransferase, ALAT Alanine Aminotransferase, CRP C-reactive protein
